# Anterior segment changes following Nd: YAG laser posterior capsulotomy: a quantitative ultrasound biomicroscopy study

**DOI:** 10.1007/s10103-025-04555-z

**Published:** 2025-06-26

**Authors:** Caner Öztürk, Selim Cevher, Mustafa Duran, Mehmet Barış Üçer, Oğuzhan Töngüş

**Affiliations:** 1 Hitit University, Çorum, Türkiye; 2 Sincan Training and Research Hospital, Ankara, Türkiye

**Keywords:** Nd:YAG laser, Capsulotomy, Anterior segment, Ultrasound biomicroscopy

## Abstract

Purpose: To evaluate the effects of neodymium-doped yttrium-aluminum-garnet (Nd: YAG) laser posterior capsulotomy on anterior segment parameters in pseudophakic eyes using ultrasound biomicroscopy (UBM).

Methods: This prospective study included 35 pseudophakic eyes of 34 patients with visually significant posterior capsule opacification (PCO) following uncomplicated phacoemulsification with a one-piece hydrophobic acrylic intraocular lens. UBM was used to assess anterior segment parameters, including anterior chamber angle (ACA), anterior chamber depth (ACD), anterior chamber width (ACW), lens vault (LV), and iris thickness (IT). The angle opening distances (AOD) at 500 μm (AOD500), and at 750 μm (AOD750), the angle recess area (ARA) at 500 μm (ARA 500), the trabecular-iris space area at 500 μm (TISA 500), and at 750 μm (TISA 750) were measured both temporal and nasal area. The measurements were taken three times, the first time before the Nd: YAG capsulotomy, the second time 1 week after and the third time 1 month after the procedure.

Results: No statistically significant changes were found in intraocular pressure (IOP), central corneal thickness, ACD, ACW, LV, or IT following capsulotomy. However, a significant and sustained increase was observed in angle-related parameters (ACA, AOD500/750, ARA500, and TISA500/750) at both 1 week and 1 month post-procedure(*p* < 0.05 for all). No significant changes were detected between the 1st week and 1st month measurements of angle-related parameters (*p* > 0.05 for all).

Conclusion: Nd: YAG laser capsulotomy leads to significant widening of anterior chamber angle structures without affecting IOP or ACD. These findings suggest that the procedure is safe and may positively influence aqueous humor dynamics.

## Introduction

Cataract is the leading cause of blindness worldwide, and cataract surgery is one of the most commonly performed surgeries across the world [1]. The most frequent complication after cataract surgery is posterior capsule opacification (PCO). Following phacoemulsification and intraocular lens (IOL) implantation, PCO is primarily caused by the proliferation, migration and adhesion of residual lens epithelial cells in the space between the posterior capsule and the IOL [2]. In addition, debris from inflammatory cells and mechanical folds in the capsular bag may contribute to the development of PCO [3].

Neodymium-doped yttrium-aluminum-garnet (Nd: YAG) laser capsulotomy is an effective procedure and the gold standard for treating PCO in pseudophakic eyes [4]. Complications such as increased intraocular pressure (IOP), corneal damage, uveitis, IOL damage, cystoid macular edema and retinal detachment have been reported following Nd: YAG laser capsulotomy. Since these complications are rare, Nd: YAG laser capsulotomy is frequently performed as it is an inexpensive, fast, noninvasive and easy-to-learn procedure [5].

Ultrasound biomicroscopy (UBM) is a high-resolution imaging technique that provides noninvasive, in vivo visualization of the structures of the anterior segment of the eye [6]. UBM provides highly detailed, two-dimensional images of various anterior segment structures and allows both quantitative and qualitative analysis [7].

Anterior chamber parameters after Nd: YAG capsulotomy can vary and conflicting results have been reported previously [8–10]. The aim of the present study was to investigate the effect of Nd: YAG laser capsulotomy on anterior chamber parameters using UBM.

## Materials and methods

This prospective study was in accordance with the tenets of the Declaration of Helsinki and had local ethics committee approval (approval number: 2022-67). Thirty-five pseudophakic eyes of 34 patients were included in the study. Written informed consent was obtained.

Patients who underwent uncomplicated phacoemulsification surgery with a hydrophobic one-piece posterior chamber IOL (AcrySof SA60; Alcon, Fort Worth, Texas, USA) and developed PCO that compromised visual acuity were included in the study. Patients with complicated cataract surgery, previous ocular surgery (except phacoemulsification) or trauma, corneal pathology, glaucoma, uveitis, pseudoexfoliation syndrome, or retinal pathology were excluded.

A detailed ophthalmic examination and measurements were performed three time points: before Nd: YAG capsulotomy treatment, 1 week after the procedure, and 1 month after the procedure. At each visit, best corrected visual acuity (BCVA), Goldmann applanation tonometry, slit-lamp biomicroscopy, fundoscopy and UBM were conducted.

All posterior capsulotomies were performed by the same ophthalmologist using a Q-switched Nd: YAG laser (Optimis Fusion, Quantel Medical) with a contact lens (Abraham YAG laser capsulotomy lens, Ocular Instruments, Inc.). After explaining the procedure and obtaining informed consent, pupils were dilated with 1% tropicamide drops (Tropamid, Bilim Pharmaceuticals, Istanbul, Turkey). A circular pattern posterior capsulotomy was then performed, sized between 4.0 and 4.5 mm, using a energy range of 0.9 to 3.0 mJ/pulse, as required to rupture the posterior capsule. After the procedure, brimonidine tartarate 0.15% (Alphagan P, Allergan, Inc., Irvine, CA, USA) eye drop was administered twice daily for 3 days and prednisolone acetate 1% (Pred Forte; Allergan, Inc., Irvine, CA, USA) eye drop was administered four times daily for one week.

UBM (Aviso A/B, Quantel Medical, MT, USA) measurements were obtained using a 50 Hz probe. After applying topical anesthetic (Proparacaine HCl 0.5%, Alcon Pharmaceuticals, Rijksweg, Belgium), a lid speculum was inserted and a saline-filled eyecup was placed. In a supine position in a dark room, participants were asked to focus on a distant target on the ceiling with their fellow eye. The probe was placed to the ocular surface and moved in small movements until the target areas were observed. Special care was taken not to press down on the globe, as this could potentially result in alterations to the angle configuration. Images were taken in the nasal and temporal quadrants while the subject fixated on a target. The radial scan pattern of the cornea was also evaluated for the central corneal thickness (CCT). All of the images were taken by the same experienced ophthalmologist. Several images were taken at each visit. The clearest image was used for measurement of anterior chamber parameters. The parameters of the anterior segment were measured quantitatively by a single examiner using a caliper integrated in the UBM software.

**Fig. 1:**
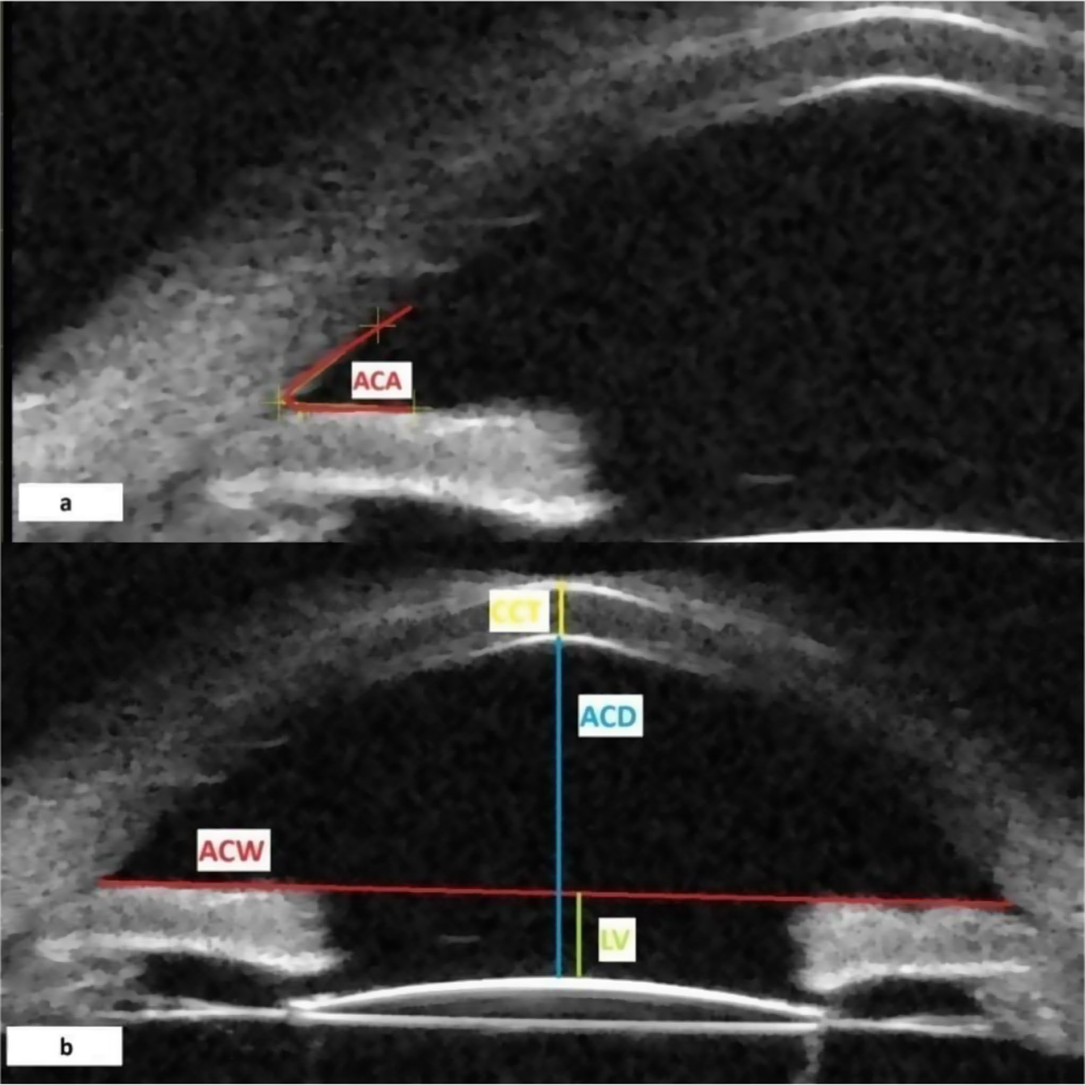
Anterior chamber angle (ACA) (**a**), central corneal thikness (CCT), anterior chamber depth (ACD), anterior chamber width (ACW), and lens vault (LV) (**b**) measurements by ultrasound biomicroscopy

**Fig. 2:**
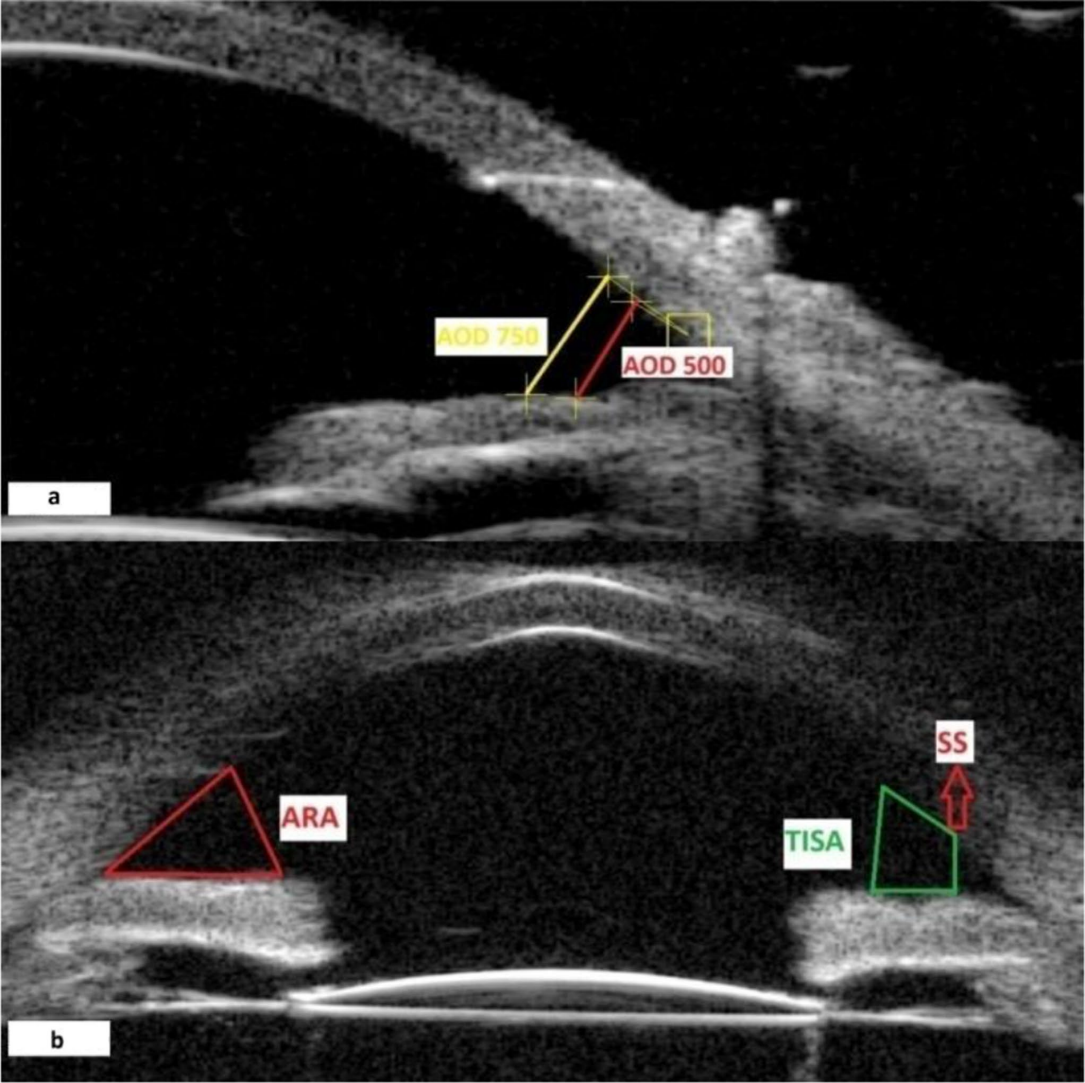
Angle opening distance at 500 and 750 μm (AOD 500, AOD 750) (**a**), angle recess area (ARA) and trabecular iris space area (TISA) (**b**) measurements by ultrasound biomicroscopy

The scleral spur was determined by the difference in tissue density between the longitudinal muscle of the ciliary body and collagen fibres of the scleral spur. The anterior segment parameters were measured according to the methods of recent studies [11–15]. The anterior chamber angle (ACA) width was obtained by measuring the angle between the iris tangential line and the posterior corneal surface with its apex in the angle recess (Fig. 1a). Anterior chamber depth (ACD) is defined as the axial distance between the corneal endothelium and the anterior pole of the IOL at the centre of the cornea. Anterior chamber width (ACW) was measured as the distance between the two scleral spurs. The perpendicular distance from the anterior pole of the IOL to the horizontal line between the scleral spurs was calculated as the lens vault (LV). (Fig. 1b) Angle opening distances (AOD) at 500 μm (AOD500) and at 750 μm (AOD750) were measured as the the distance between the posterior corneal surface and the anterior iris surface on a line perpendicular to the trabecular meshwork 500 μm and 750 μm from the scleral spur. (Fig. 2a) Angle recess area (ARA) at 500 μm (ARA 500) is defined as an area of the angle recess bounded anteriorly by the AOD 500. Trabecular-iris space area at 500 μm (TISA 500) and at 750 μm (TISA 750) were defined as the area bounded anteriorly by the AOD 500 and AOD 750, respectively; posteriorly by a line drawn from the scleral spur perpendicular to the plane of the inner scleral wall to the opposing iris; inferiorly by the iris surface; and superiorly by the inner corneoscleral wall. (Fig. 2b) The iris thickness (IT) at 750 μm (IT 750) was defined as the thickness of the iris at 750 μm from the scleral spur. The linear parameters were given in millimetres and the angular parameters were given in degrees.

### Statistical analysis

SPSS (IBM Corp., Armonk, NY, USA) version 22 software was used for data analysis. Analytical methods (Kolmogorov-Smirnov/Shapiro-Wilk tests) were used to assess whether the data were normally distributed. Normally distributed data were compared using analysis of variance (ANOVA) for repeated measures. The Greenhouse-Geisser correction was applied if the sphericity assumption was not met. Non-normally distributed data were evaluated using the Friedman test and pairwise comparisons were evaluated using the Wilcoxon test with post-hoc Bonferroni correction. Data are expressed as mean and standard deviation (SD). *P* < 0.05 was considered significant.

## Results

This study was performed with 35 pseudophakic eyes of 34 patients. The mean age of the patients was 66.3 ± 3.67 (range 59–73). Table 1 shows the changes in IOP, CCT, ACD, ACW, LV, and IT values before and after Nd: YAG capsulotomy. There was no statistically significant change in IOP (*p* = 0.821) and CCT (*p* = 0.456) before and after the Nd: YAG capsulotomy. Anterior chamber depth and ACW showed a minimal increase from baseline to one month post procedure, but this change was not statistically significant (*p* = 0.502, and *p* = 0.790 respectively). Lens vault and IT did not demonstrate significant alterations after Nd: YAG capsulotomy (LV: *p* = 0.349; IT nasal: *p* = 0.777; IT temporal: *p* = 0.568).

Changes in angle-related parameters before and after Nd: YAG laser posterior capsulotomy showed in Table 2. A significant widening of the ACA was observed both nasally and temporally after Nd: YAG laser capsulotomy (*p* = 0.001, for both). The nasal AOD 500 and AOD 750 (*p* = 0.02, for both) and the temporal AOD 500 and AOD 750 (*p* < 0.01, for both) were increased after the Nd: YAG capsulotomy. The ARA 500 and TISA 500 increased significantly after Nd: YAG capsulotomy in both the nasal and temporal quadrants (*p* < 0.001, for both). Similarly, the TISA 750 showed a significant increase after the laser procedure (*p* < 0.001 nasal and *p* = 0.001 temporal).

Post hoc analysis revealed that anterior chamber parameters, including ACA, AOD 500, AOD 750, ARA 500, TISA 500, and TISA 750, showed significant increases at 1 week after Nd: YAG laser capsulotomy, with stabilization by the 1-month follow-up. No significant changes in angle-related parameters were observed between 1 week and 1 month after the laser procedure (*p* > 0.05, for all).

**Table 1:** The IOP, CCT, ACD, ACW, LV, and IT values before and after nd: YAG capsulotomy

	Before Nd: YAG capsulotomy (mean ± SD)	1 week after Nd: YAG capsulotomy (mean ± SD)	1 month after Nd: YAG capsulotomy (mean ± SD)	*P*
IOP (mm-Hg)	16.83 ± 2.43	16.94 ± 2.52	16.43 ± 2.34	0.821^a^
CCT (µm)	557.5 ± 56.4	552.9 ± 54.4	555.9 ± 57.6	0.456^a^
ACD (mm)	3.92 ± 0.28	3.90 ± 0.30	4.01 ± 0.22	0.502^a^
ACW (mm)	10.97 ± 0.57	11.07 ± 0.54	11.15 ± 0.61	0.790^f^
LV (mm)	1.18 ± 0.23	1.14 ± 0.27	1.19 ± 0.24	0.349^a^
IT (nasal) (mm)	0.47 ± 0.09	0.48 ± 0.08	0.47 ± 0.11	0.777^a^
IT(temporal)(mm)	0.49 ± 0.10	0.47 ± 0.06	0.51 ± 0.10	0.568^a^

*Abbrevations* IOP: intraocular pressure; CCT: central corneal thickness; ACD: anterior-chamber depth; ACW: anterior-chamber width; LV: lens vault; IT: iris thickness; f: Friedman test; a: Analysis of variance (ANOVA)

**Table 2:** Changes in angle-related parameters before and after nd: YAG laser posterior capsulotomy

	Before Nd: YAG capsulotomy (mean ± SD)	1 week after Nd: YAG capsulotomy (mean ± SD)	1 month after Nd: YAG capsulotomy (mean ± SD)		*P*	P1	P2	P3
**Nasal**								
ACA (degree)	42.50 ± 4.18	45.21 ± 4.50	45.62 ± 4.73	0.001^a^		0.004^w^	0.001^w^	0.180^w^
AOD 500 (mm^2^)	0.46 ± 0.07	0.54 ± 0.06	0.55 ± 0.08	0.002^f^		0.002^w^	< 0.001^w^	0.938^w^
AOD 750 (mm^2^)	0.82 ± 0.12	0.93 ± 0.12	0.91 ± 0.12	0.002^f^		0.002^w^	< 0.001^w^	0.943^w^
ARA 500 (mm^2^)	0.15 ± 0.03	0.20 ± 0.04	0.19 ± 0.04	< 0.001^a^		< 0.001^w^	< 0.001^w^	0.484^w^
TISA 500 (mm^2^)	0.27 ± 0.04	0.32 ± 0.05	0.32 ± 0.06	< 0.001^a^		< 0.001^w^	< 0.001^w^	0.632^w^
TISA 750 (mm^2^)	0.49 ± 0.08	0.58 ± 0.09	0.57 ± 0.09	< 0.001^a^		< 0.001^w^	< 0.001^w^	0.853^w^
**Temoral**								
ACA (degree)	42.70 ± 5.32	45.65 ± 6.09	45.74 ± 6.12	0.001^f^		0.001^w^	< 0.001^w^	0.134^w^
AOD 500 (mm^2^)	0.47 ± 0.09	0.54 ± 0.09	0.55 ± 0.09	< 0.001^a^		< 0.001^w^	< 0.001^w^	0.194^w^
AOD 750 (mm^2^)	0.82 ± 0.12	0.91 ± 0.14	0.90 ± 0.11	< 0.001^a^		< 0.001^w^	< 0.001^w^	0.385^w^
ARA 500 (mm^2^)	0.16 ± 0.04	0.20 ± 0.04	0.19 ± 0.04	< 0.001^a^		< 0.001^w^	< 0.001^w^	0.346^w^
TISA 500 (mm^2^)	0.28 ± 0.04	0.32 ± 0.06	0.33 ± 0.06	< 0.001^f^		< 0.001^w^	< 0.001^w^	0.220^w^
TISA 750 (mm^2^)	0.52 ± 0.09	0.58 ± 0.10	0.58 ± 0.09	0.001^a^		0.001^w^	0.006^w^	0.084^w^

**Abbreviations**: ACA: anterior-chamber angle; AOD500: angle opening distance 500 μm anterior to the scleral spur; AOD750: angle opening distance 750 μm anterior to the scleral spur; ARA 500: angle recess area at 500 μm; TISA 500: trabecular-iris space area at 500 μm; TISA 750: trabecular-iris space area at 750 μm; f: Friedman test; a: Analysis of variance (ANOVA); w: Wilcoxon test

P1 indicates the P value between before the Nd: YAG capsulotomy and 1 week after Nd: YAG capsulotomy, P2 indicates the P value between before the Nd: YAG capsulotomy and 1 month after Nd: YAG capsulotomy, P3 indicates the P value between 1 week after Nd: YAG capsulotomy and 1 month after Nd: YAG capsulotomy

## Discussion

Posterior capsule opacification is the most common long-term complication after cataract surgery, and Nd: YAG laser capsulotomy is the gold standard for its treatment [2, 4]. Despite its effectiveness, the effects of Nd: YAG capsulotomy on anterior segment parameters have not been fully elucidated.

Previous studies have shown that IOP can vary after Nd: YAG capsulotomy. Many studies have reported transient IOP elevation after Nd: YAG capsulotomy due to the release of capsular debris and inflammatory mediators [16–18]. It has been shown that IOP elevation can be prevented with anti-glaucomatous treatment initiated before or after Nd: YAG capsulotomy [19]. In our study, there was no change in IOP 1 week and 1 month after Nd: YAG capsulotomy compared to the baseline. As we administered brimonidine tartrate prophylactically to all patients for 1 week and did not remeasure IOP in the early period, we may not have detected any change in IOP.

There are conflicting results on the change in CCT after Nd: YAG laser capsulotomy. Oztas et al. reported a significant 10-µm decrease in CCT 1 month after Nd: YAG posterior capsulotomy [20]. Pekel et al. showed that the mean CCT increased one hour after capsulotomy and then returned to baseline values at the first week and first month visits [3]. Wróblewska-Czajka et al. showed that there was a statistically significant correlation between the increase in CCT and the total laser energy used. A transient increase in CCT was found up to 3 months after laser capsulotomy [21]. Release of inflammatory mediators, IOP fluctuations, laser beam focusing errors, use of gel during the procedure, and transient endothelial cell dysfunction due to acoustic wave emission may cause a transient increase in corneal thickness after laser capsulotomy [22]. In our study, there was no change in CCT after Nd: YAG laser capsulotomy as reported in some other studies [23, 24].

Conflicting results have been published about the change in ACD after Nd: YAG capsulotomy. Some studies have reported a decrease in ACD after Nd: YAG posterior capsulotomy [20, 25]. The decreased ACD was attributed to the posterior push of the prolapsed vitreous, whereas the increased ACD after YAG capsulotomy was explained by the posterior displacement of the IOL. Eliacik et al. showed a mean posterior movement of the IOL of 60 microns after YAG capsulotomy and reported a significant increase in ACD [10]. Findl et al. reported a backward movement of the IOL of an average of 35 microns for plate haptics and an average of 18 microns for 1-piece PMMA and 3-piece foldable IOLs and showed a significant increase in ACD [26]. While some studies suggest a potential posterior shift of the IOL leading to deepening of the ACD, our study found no significant changes in LV. Similar to some other studies [24, 27], no significant change in ACD was found after Nd: YAG capsulotomy. The lack of significant changes in ACD and LV suggests that IOL position remains relatively stable in eyes with modern one-piece hydrophobic acrylic IOLs, minimising concerns about refractive shifts.

Our study is the first to evaluate the IT and ACW after Nd: YAG capsulotomy. There was no significant change in IT and ACW after Nd: YAG capsulotomy. As the pupil was dilated before the procedure and the iris was removed from the laser treatment area, there may have been no change in IT.

Our results demonstrated a significant increase in ACA, AOD 500, AOD 750, ARA 500, TISA 500, and TISA 750 at both 1 week and 1 month after Nd: YAG capsulotomy. It was observed that the angle-related parameters, which increased 1 week after the capsulotomy, remained consistent at the one-month follow-up. A study by Eliaçik et al. in 43 patients with anterior segment optical coherence tomography (AS-OCT) showed that ACA, AOD 500 and AOD 750 increased significantly after YAG capsulotomy [10]. The increases in angle-related parameters could be due to the mechanical effect of the shock waves produced by the laser on the zonules, leading to zonular weakening and IOL displacement to vitreous cavitation. Another study on patients with pseudoexfoliation (PEX) by Eliacik et al. supported this theory. In this study, YAG capsulotomy was performed in 25 eyes of 25 pseudophakic patients with PEX and 26 eyes of 26 pseudophakic control patients without PEX. In both eyes with PEX and control eyes, the nasal and temporal ACA, AOD500 and AOD750 values analysed by AS-OCT showed a significant increase after capsulotomy. After Nd: YAG laser capsulotomy, the ACA depth and width increased more in pseudophakic eyes with PEX than in control eyes [28]. On the other hand, Pekel et al. found no significant change in the ACA after YAG capsulotomy using corneal topography [3]. The discrepancy among studies could be attributed to differences in imaging modalities, laser energy levels, timing of measurements, and IOL designs that were used. Our study demonstrated that while there was no significant change in ACD and LV, angle-related parameters increased after the Nd: YAG capsulotomy. Although the changes in ACD and LV were not statistically significant, an average increase of 9 mm in ACD and 1 mm in LV was observed one month after Nd: YAG capsulotomy. The fact that the increase in LV is much more limited than ACD supports the hypothesis that the iris and IOL move together posteriorly towards the vitreous cavity after capsulotomy. The widening of angle-related parameters could be explained by posterior displacement of the iris. Our finding of a significant widening of angle-related parameters without a significant change in LV may also indicate an isolated posterior movement of the iris root rather than the entire lens-iris diaphragm due to reduced zonular stress after capsulotomy.

There are limited studies on the change in TISA 500 and TISA 750 values after yag capsulotomy. Similar to our study, el-Haddad et al. reported a significant increase in TISA 500 and TISA 750 after Nd: YAG capsulotomy [24]. To the best of our knowledge, our study is the first to examine the effect of YAG capsulotomy on ARA500 and showed that ARA500 increased significantly after Nd: YAG capsulotomy. The increase in angle-related parameters after Nd: YAG capsulotomy may have potential implications in glaucoma management. YAG capsulotomy can help to improve aqueous outflow in patients with narrow angles or early angle-closure disease.

Our study has several strengths, including the use of UBM, which allows high-resolution imaging of anterior segment structures, and standardised assessment at multiple time points. It is also the first study to examine the change in ARA 500, IT and ACW after YAG capsulotomy. However, some limitations should be acknowledged. The sample size was relatively small and the follow-up time was one month. More reliable results can be obtained from a study that follows a larger number of subjects over a longer period of time. A single examiner measured the anterior segment parameters from the clearest image. The reliability of the results would be increased by averaging measurements from three or more images. A further limitation is the absence of early post-laser imaging (e.g. within 24–48 h), which might have captured transient changes not evident at one-week or one-month follow-up.

In conclusion, our study demonstrates that Nd: YAG laser capsulotomy results in a significant and stable widening of anterior chamber angle-related parameters, including ACA, AOD, ARA and TISA, without significantly altering ACD, LV or IOP. These findings reinforce the safety of Nd: YAG capsulotomy in pseudophakic eyes and highlight its role in potentially improving aqueous humour dynamics. Future studies with larger cohorts and longer follow-up are needed to further investigate the long-term effects of these anatomical changes, especially in glaucoma patients or those with compromised anterior segment anatomy. Also future studies can obtain more reliable and comparable results by using AS-OCT and UBM together.

## Data Availability

The data that support the findings of this study are available from the corresponding author upon reasonable request.
